# Embedded word priming elicits enhanced fMRI responses in the visual word form area

**DOI:** 10.1371/journal.pone.0208318

**Published:** 2019-01-10

**Authors:** Zhiheng Zhou, Carol Whitney, Lars Strother

**Affiliations:** 1 Department of Psychology, University of Nevada, Reno, NV, United States of America; 2 Independent Researcher, Silver Spring, MD, United States of America; University of Valencia, SPAIN

## Abstract

Lexical embedding is common in all languages and elicits mutual orthographic interference between an embedded word and its carrier. The neural basis of such interference remains unknown. We employed a novel fMRI prime-target embedded word paradigm to test for involvement of a visual word form area (VWFA) in left ventral occipitotemporal cortex in co-activation of embedded words and their carriers. Based on the results of related fMRI studies we predicted either enhancement or suppression of fMRI responses to embedded words initially viewed as primes, and repeated in the context of target carrier words. Our results clearly showed enhancement of fMRI responses in the VWFA to embedded-carrier word pairs as compared to unrelated prime-target pairs. In contrast to non-visual language-related areas (e.g., left inferior frontal gyrus), enhanced fMRI responses did not occur in the VWFA when embedded-carrier word pairs were restricted to the left visual hemifield. Our finding of fMRI enhancement in the VWFA is novel evidence of its involvement in representational rivalry between orthographically similar words, and the co-activation of embedded words and their carriers.

## Introduction

Human ventral occipital temporal cortex (vOT) is frequently implicated in visual object recognition and reading. A “visual word form area” (VWFA; [1, 2]) in left vOT exhibits differentiable fMRI responses to words as compared to pseudowords and non-linguistic control stimuli [3–8]. The VWFA is highly sensitive to orthographic structure [2, 9–15]. This sensitivity is presumably the result of extensive experience [10, 16, 17], and it is consistent with the possibility that the VWFA serves as an interface between orthography-sensitive visual representations and those in non-visual language centers of the human brain.

The purpose of the current study was to measure the sensitivity of the VWFA and other regions of the brain to lexical embedding using fMRI. Lexical embedding is common in English [18]. Unlike other words, embedded words elicit a unique type of representational rivalry in which an embedded word and its carrier compete for both orthographic and semantic representation within the word recognition circuit (e.g., ‘CAR’ in ‘CART’). Embedded-carrier rivalry is the result of mutual interference at different levels of form-to-meaning representation during cascaded orthographic and semantic processing, which begins at the level of orthography [19, 20]. By virtue of their inherent orthographic similarity, embedded words and their carriers co-activate and therefore compete for representation via mutual inhibition [21]. Traditionally, orthographic neighbors refer to similar words with one letter substitution. However, previous studies have shown that the embedded word and its carrier are also orthographic neighbors by letter deletion and addition, respectively, and exhibit orthographic similarity interference effects [22–24]. The neural basis of representational rivalry during embedded word viewing is currently unknown. Given its sensitivity to orthography, we predicted the VWFA as a highly plausible candidate, in addition to other brain regions involved in cascaded orthographic-semantic mapping. We tested our prediction using a novel embedded prime-target word pair paradigm, in two fMRI experiments. In the scanner, observers viewed three-letter primes (the embedded word) followed by four-letter targets (the carrier word) which either contained the prime (‘CAR’ → ‘CART’ or ‘CAR’ → ‘SCAR’), or shared no letters (‘CAR’ → ‘STEM’).

We hypothesized three possible outcomes of our fMRI experiments: repetition *enhancement* [25–27], repetition *suppression* [10, 28–30], or both. Findings of mutual interference between the embedded words and their carriers in behavioral priming studies indicate a co-activation of the embedded words and their carriers [22, 23]. Such co-activation supports the possibility of repetition enhancement of fMRI responses, which are typically consistent with interference effects underlying negative priming [31–33]. The observation of fMRI repetition enhancement in the VWFA would be the first of its kind. A potentially related finding was recently reported in left vOT (in a location consistent with the VWFA) for syllabic negative priming [25]. On the other hand, fMRI repetition suppression has been proposed to reflect a facilitation effect due to repetition priming. Specifically, the VWFA showed repetition suppression for repeated whole words (‘CART’ → ‘CART’) but not for repeated sublexical orthographic structure (‘CART’ → ‘CAST’), in an fMRI priming study [10]. Because embedded words followed by their carriers (e.g., ‘CAR’ → ‘CART’) share letters that form the embedded word, this could result in repetition suppression in the VWFA for these kinds of word pairs. This possibility is consistent the results of the Glezer et al. study [10], in which equivalent release from suppression occurred for one letter substitution (‘CART’ → ‘CAST’) and whole word change (‘CART’ → ‘STEM’), neither of which involve lexical repetition, despite sub-lexical repetition in the first case (i.e., three of the four letters are repeated between the word pairs). Additionally, the suppression prediction is also supported by the results of Devlin et al. [34], which demonstrated repetition suppression in left vOT (in a location consistent with the VWFA) underlying orthographic priming during the viewing of orthographically similar words with lexical embedding under conditions of letter subtraction (e.g., ‘passive’ → ‘PASS’). Taken together with the results of the study by Glezer et al., it is reasonable to predict suppressed fMRI responses to embedded word repetition in our study, despite differences in letter addition as opposed to substitution (Glezer et al.,) and subtraction (Devlin et al.).

It should be noted that fMRI repetition enhancement and suppression are not mutually exclusive, and could plausibly co-occur and cancel out. For instance, it is possible that for embedded word pairs, both enhancement and suppression co-occur in the same orthography-sensitive brain region (e.g., the VWFA), either simultaneously or at different latencies. In either case, canceling out could occur, because although the latency of repetition suppression voxels is typically faster than the latency of repetition enhancement voxels (~3 s); due to poor temporal resolution of fMRI and the limitation of the current study, it would be impossible to detect such a difference [35]. Additionally, or alternatively, concurrent enhancement and suppression for the embedded words could co-occur in different brain regions based on sensitivity to visual versus language-related characteristics of stimuli (e.g., [27]).

While left vOT was of primary interest, the VWFA in particular, we also anticipated the involvement of left inferior frontal gyrus (IFG) given its involvement in both orthographic and semantic processing underlying word recognition. For example, using an fMRI repetition suppression paradigm similar to that adopted here, Glezer et al. [36] showed that the left IFG was associated with release of fMRI repetition suppression (i.e. an absence of suppression due to change) for homophones and different words as compared to repeated words, indicating a sensitivity to orthography in the left IFG. Another fMRI study by Purcell, Jiang, & Eden [37] also found that the left IFG is sensitive to orthography and works together with left vOT during reading and spelling. The authors suggested that there are three possible roles of the left IFG, which might map orthographic and phonological representations, involve orthographic long-term memory, or handle competition for multiple lexical units. Consistent with this view, a study by Pas et al. [25] found repetition enhancement for syllabically similar prime-target pairs as compared to unrelated pairs. The authors interpreted their results as evidence that the left IFG is involved in resolving lexical competition between similar words by mediating co-activated lexical neighbors, which compete via mutual inhibition for representation. Taken together, these findings support the possibility of observing that the left IFG will show responses to embedded words.

Finally, in addition to our primary manipulations of prime and target, we tested for a prospective effect of hemifield by presenting prime-target word pairs in either the right hemifield (RVF → RVF), left hemifield (LVF → LVF), or by varying the location of the prime and target between hemifields (LVF → RVF or RVF → LVF). Early fMRI studies showed location-invariant word representation in the VWFA [2, 38, 39], but this finding has since been challenged by findings of position sensitivity in the VWFA and other portions of left vOT [40–45]. There are two general predictions related to the hemifield effect in the VWFA. If the VWFA is location-invariant, we predict that repetition enhancement (or conversely, suppression) will be the same for all conditions; if the VWFA is position sensitive, we predict that the neural responses will be maximal in the RVF-RVF condition, which is consistent with the contralateral bias of orthography-sensitive mechanisms in left vOT, but that it will nevertheless occur in the remaining conditions.

## Methods

### Participants

Eleven right-handed observers (3 females and 8 males; mean age 33.1 years, range 25–47 years) participated in both Experiments 1 and 2. All observers were right-handed literate native English speakers with normal or corrected-to-normal vision, and none of them had neurological or psychiatric disorders. All participants were recruited from the University of Nevada, Reno, and the study with all consent forms and experimental procedures was approved by the Institutional Review Board of University of Nevada, Reno.

### Stimulus apparatus

All experiments were conducted using a 2.53 GHz MacBook Pro with an NVIDIA GeForece 330 M graphics processor (512 MB of DDR3 VRAM). Stimuli were created and presented using PsychotoolBox-3 [46, 47] for MATLAB (The MathWorks Inc., Natick, MA). Observers viewed stimuli through a mirror attached to the head coil which projected a 32 in. SensaVue (1920 × 1080 resolution; 31.5° × 18.9° visual angle; 85 Hz refresh rate) visual display system (Invivo, Inc., Gainesville, FL) ~125 cm anyway outside of the scanner bore.

### Main experiments

**Fig 1:**
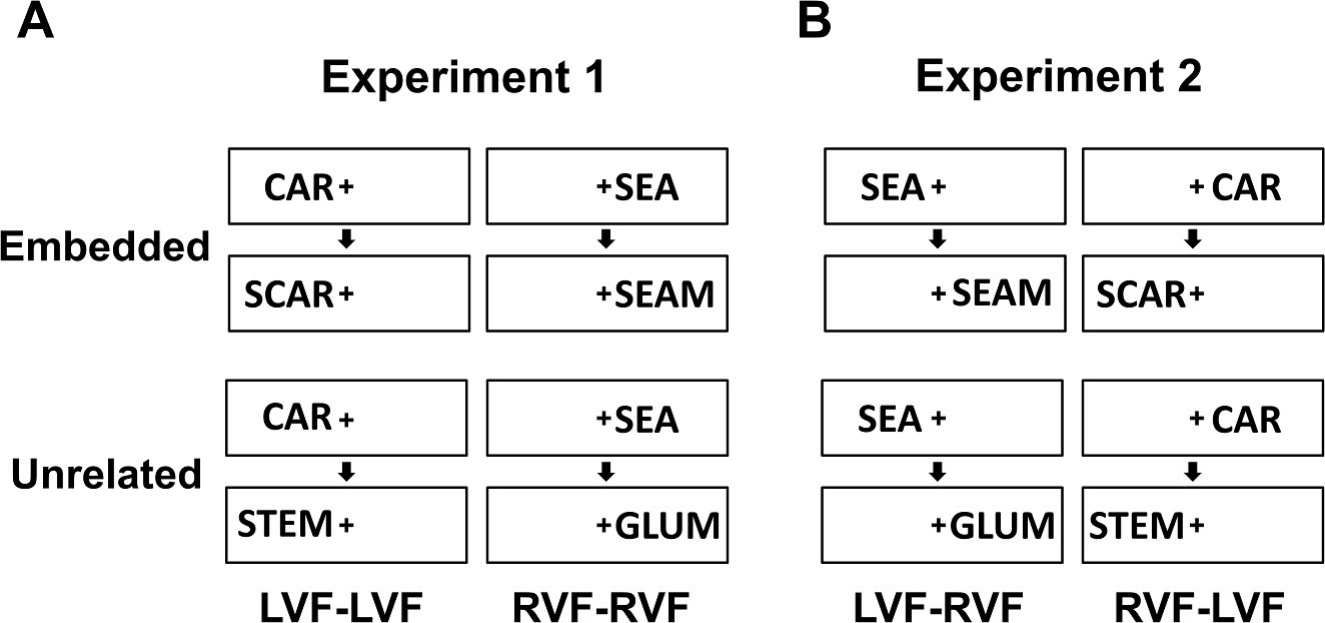
Stimuli and conditions in Experiment 1 and 2. In the *embedded* condition, the target repeats the primed embedded word and adds a letter to the prime; in the *unrelated* condition, the target shares no letter with the prime. (A) In Experiment 1, the prime either appears in the LVF or in the RVF, and the target always appears in the same hemifield as the prime. (B) In Experiment 2, the prime either appears in the LVF or in the RVF, and the target always appears in the opposite hemifield as the prime.

We used a rapid event-related fMRI prime-target design in which primes were three-letter words followed by four-letter word targets, in two separate experiments. Each experiment employed a 2 × 2 factorial design (Fig 1A and 1B) with independent variables of embeddedness and prime-target hemifield location(s). In the *embedded* condition, primes were embedded within the target carrier word (e.g. CAR-CART or CAR-SCAR), and in the *unrelated* condition, the target shared no letter with the prime (e.g. CAR-STEM). In Experiment 1, the prime and target were always presented in the same visual hemifield (i.e. both prime and target in LVF or RVF). In Experiment 2, the prime and target were always presented in opposite hemifields (i.e. prime in LVF and target in RVF, or prime in RVF and target in LVF).

The procedures for both experiments were identical. Each experiment started with a central fixation cross followed by a variable inter-trial-interval (ITI), which ranged between 3 and 11 s (in 2 s increments), and the appearance of a three-letter prime in either the LVF or RVF, always for 0.3 s. Four-letter target words appeared 0.4 s after the prime had disappeared, either in same visual hemifield as the prime (Experiment 1), or in the opposite visual hemifield (Experiment 2), always for 0.3 s. Targets and their embedded primes were never morphologically related. The morphological relation between the prime and target was defined according to the study of Devlin et al. [34], in which “morphologically related” means that the prime and target contain the same orthographic structure as well as the same semantic meaning. Accordingly, PASSIVE → PASS are orthographically but not semantically related, and thus are not morphologically related. However, HUNTER → HUNT are both orthographically and semantically related and thus morphologically related. All words were displayed in black against a gray background. The fixation cross subtended a visual angle of 0.2° × 0.2°, and four-letter words subtended an angle of 3.4° × 0.8°. The inner edge of each word fell just next to the fixation cross. To maintain the same visual overlap between the prime and target across hemifields, the additional letter in the target was in the first position in the LVF and in the fourth position in the LVF (Fig 1).

Each experiment consisted of 4 runs and all 8 runs were collected in the same scan session with each experiment run alternating after the other. Observers viewed a total of 224 different English prime-target pairs in each experiment (All words are listed in S1 Table). First-letter addition and last-letter addition trials each contained 112 prime-target pairs. Due to the limited availability of stimulus pairs, the psycholinguistic variables for word pairs were not fully controlled. To avoid possible confounding variables due to this limitation, the word stimuli in *LVF-LVF* (Experiment 1) and *RVF-LVF* (Experiment 2) conditions, and the *RVF-RVF* (Experiment 1) and *LVF-RVF* (Experiment 2) conditions were the same and randomized across runs for the *embedded* and *unrelated* conditions. Each run started and ended with a 10-s fixation, and there were 14 trials per each condition with a total of 56 trials which lasted for 360 s. Trial sequences and ITIs were generated using the Optseq (https://surfer.nmr.mgh.harvard.edu/optseq) to optimize the rapid event-related fMRI design. Observers maintained central fixation throughout the entire experiment. A change-detection fixation task was used to encourage and monitor central fixation (observers pressed a button when the fixation cross changed from black to gray). Changes in the fixation cross occurred randomly for 50% of the ITIs.

### Localizer scans

We identified the VWFA for each individual observer using a standard block design localizer experiment. This experiment was performed separately from the main experiments. Stimulus were 2-D grayscale (~5° × 5° for non-word stimulus; ~5° × 1° for words) images presented centrally against a white background consisting of words, faces, common daily objects, or scrambled images. Each block was presented for 16 seconds, and within each block, 16 images of the same category were presented for 0.5 s followed by 0.5 s blank screen. There were 16 blocks in each run and 4 blocks per each stimulus category, and the block order was counterbalanced across runs. 9 out of 11 observers completed 2 runs, and the other 2 observers completed 1 run due to scheduling conflicts. The region of interest (ROI) of VWFA was defined for each observer as a cluster of voxels (*p* < 0.05 ~ 0.01, uncorrected) in which the BOLD responses were greater for words compared with scrambled images. The VWFA was constrained to clusters that showed responses in anatomical landmark regions consisting of the fusiform gyrus and inferior occipitotemporal sulcus. The same contrast was also used to define the ROI of left IFG for 10 out of 11 observers, and the Word > Fixation contrast was used to identify the left IFG for the last observer who did not show any activation in the left IFG using the Word > Scrambled image contrast. The left IFG was constrained to correspond the location of the left IFG which was known for orthographic processing [36, 37].

### fMRI data acquisition

The main experiments and the localizer scans were conducted at the Renown Health hospital (Reno, NV) using a 3T Philips Ingenia MRI system equipped with a 32-channel digital SENSE head coil. Continuous whole-brain BOLD signals were collected using T2*-weighted interleaved, echo-planar functional images (TE = 40 ms, TR = 2 s, flip angle = 71°, 32 axial slices, 3 mm^2^, 2 mm thickness, 1 mm gap, matrix size = 128 × 128, field of view = 240 × 240). Dummy scans were collected for a minimum of 10 s at the beginning of every run to allow for stabilization of the magnetic field. High-resolution anatomical images obtained using a 3-D T1-weighted pulse sequence (TE = 4.60 ms, TR = 3.0 s, flip angle = 8°, resolution = 1 × 1 × 1 mm, matrix size = 256 × 256) and were used for anatomical reconstruction of the cortical hemisphere surfaces.

### fMRI data preprocessing

Data were preprocessed and analyzed using AFNI [48], SUMA [49], FreeSurfer [50, 51], and MATLAB. We performed corrections for slice scan time and head motion (always < 2 mm), and each functional voxel was temporally normalized using AFNI’s 3dDetrend command. Functional data were spatially smoothed using a Gaussian kernel of 6 mm. The group level analyses of the data were based on (1) a whole-brain analysis using the 3dANOVA3 function for standardized Talairach space [52] data and (2) a ROI analysis using an independent localizer (described earlier) in which the anatomical volume was transformed to surface for defining the surface-based topographic ROIs.

### fMRI data analysis

Statistical analyses based on the general linear model (GLM) were performed on each voxel to obtain beta weights (coefficients) by convolving with a model hemodynamic response function using a BLOCK model in AFNI’s 3dDeconvolve function. Nine additional nuisance regressors were also included: three run-wise baseline parameters corresponding to constant signal, linear drift, and second-degree polynomials, and six rigid motion registration parameters.

For the group-level whole-brain GLM, each individual’s data was first transformed into standard Talairach space. Statistical maps were calculated based on the 2 × 2 factorial model using AFNI’s 3dANONVA3 function which accounted for both within- and between-participant variance. The statistical threshold was set at voxel-wise *p* < 0.01 with cluster size larger than 28 voxels at *p* < 0.05 cluster-level corrected, determined by the AFNI AlphaSim function with Monte Carlo simulations. To increase the statistical power for the effect on embeddedness, we performed additional analysis by collapsing the prime-target hemifield and combining two experiments (8 runs total), and the statistical maps were calculated based on a one factorial model for the embeddedness. The statistical threshold was set at voxel-wise *p* < 0.005 with cluster size larger than 23 voxels at *p* < 0.01 cluster-level corrected, determined by the AFNI AlphaSim function with Monte Carlo simulations.

For the group-level ROI analyses, a separate general linear model was applied using the combined data of all 4 four runs for each experiment in which the finite impulse responses were derived for each condition staring from 4 s prior to and extending 20 s following the start of each events (TENT model in AFNI’s 3dDeconvolve function). BOLD time-courses were derived for all four conditions based on average BOLD signals in all voxels within each ROI. A 2 × 2 repeated-measures ANOVA was conducted on the peak BOLD responses, corresponding to the 6 and 8 s, for embeddedness (*Embedded* and *Unrelated*) and prime-target hemifield location(s) (Experiment 1: *LVF-LVF* and *RVF-RVF*; Experiment 2: *LVF-RVF* and *RVF-LVF*).

## Results

### Whole-brain analyses

**Fig 2:**
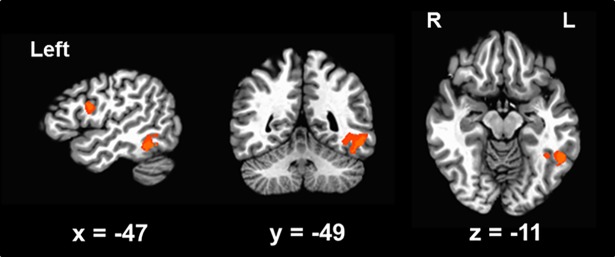
Results of whole brain analyses from combining Experiment 1 and 2. The effect of embeddedness is revealed by the *Embedded* > *Unrelated* contrast, which yields enhanced fMRI responses in the VWFA and left IFG.

We performed whole-brain group-level analyses combining the two experiments (see more details in Method session). Fig 2 and Table 1 show the results from this whole-brain analyses. First, we sought to identify clusters showing greater responses for embedded words by the contrast *embedded* > *unrelated*. We observed that embedded words was associated with brain responses in the left fusiform gyrus (FG), left precuneus (PCun), left IFG and left middle frontal gyrus (MFG). The center of mass of left FG was located in the Talairach coordinate, x = -47, y = -49, z = -11, which corresponded to the known VWFA location [2, 10, 53]. Second, we aimed to find any brain regions that might show repetition suppression related to the embedded words processing by the contrast *unrelated* > *embedded*. However, no cluster showed repetition suppression even at a relatively liberal threshold (*p* < 0.01, uncorrected). These results indicated the effect of whole-word embedding was associated with fMRI repetition enhancement, but there was no fMRI repetition suppression associated with embedded words.

**Table 1:** Results of the significant activations revealed by the whole-brain analysis.

Contrast	Brain Region	Hemisphere	Cluster Center Coordinates			Number of Voxels	Threshold (Cluster Corrected)
			x	y	z		
**Experiment 1 and 2 combined**	** **	** **	** **	** **	** **	** **	** **
***Embedded* > *Unrelated***	**Fusiform gyrus**	**Left**	**-47**	**-49**	**-11**	**76**	***p* < 0.005**
** **	**Precuneus**	**Left**	**-24**	**-69**	**34**	**57**	***p* < 0.005**
** **	**Inferior frontal gyrus**	**Left**	**-46**	**4**	**21**	**52**	***p* < 0.005**
** **	**Middle frontal gyrus**	**Left**	**-42**	**30**	**23**	**27**	***p* < 0.005**
**Experiment 1**	** **	** **	** **	** **	** **	** **	** **
***Embedded* > *Unrelated***	**Middle frontal gyrus**	**Left**	**-41**	**30**	**23**	**42**	***p* < 0.01**
** **	**Precuneus**	**Left**	**-24**	**-69**	**33**	**37**	***p* < 0.01**
** **	**Cerebellum**	**Left**	**-14**	**-42**	**-16**	**32**	***p* < 0.01**
** **	**Fusiform gyrus**	**Left**	**-47**	**-47**	**-16**	**30**	***p* < 0.01**
** **	**Inferior frontal gyrus**	**Left**	**-43**	**4**	**20**	21*	***p* < 0.01**
** **	**Thalamus**	**Right**	**11**	**-13**	**0**	**37**	***p* < 0.01**
**Experiment 2**	** **	** **	** **	** **	** **	** **	** **
***Embedded* > *Unrelated***	**Fusiform gyrus**	**Left**	**-47**	**-49**	**-11**	**92**	***p* < 0.01**
** **	**Inferior frontal gyrus**	**Left**	**-43**	**1**	**24**	**44**	***p* < 0.01**
** **	**Precuneus**	**Left**	**-27**	**-67**	**26**	15*	***p* < 0.01**

Note

* Cluster-level uncorrected

We then performed separate whole-brain group-level analyses based the 2 × 2 factorial design for each experiment. Table 1 shows the whole-brain analyses results for Experiment 1 and 2. In Experiment 1, we observed similar results of embeddedness as in the combined analysis, in which the *Embedded* > *Unrelated* contrast revealed activation in the left FG, left PCun, and left IFG (Note: it did not survive after cluster-level correction). Again, there was no significant voxel revealed by the *Unrelated* > *Embedded* contrast. At a liberal threshold (*p* < 0.05, uncorrected), we observed repetition enhancement in the left FG and left IFG in the *RVF-RVF* condition, however, only the left IFG was associated with repetition enhancement in the *LVF-LVF* condition. In Experiment 2, we again observed repetition enhancement in the same brain regions, including the left FG, left IFG, and left PCun (Note: it did not survive after cluster-level correction), revealed by the *Embedded* > *Unrelated* contrast. Similarly, no brain region showed suppression as defined by the *Unrelated* > *Embedded* contrast. At a liberal threshold (*p* < 0.05, uncorrected), unlike Experiment 1, both the *LVF-RVF* and *RVF-LVF* condition yielded significant repetition enhancement in the left FG and left IFG. In short, analyses for both experiments showed consistent results that an enhancement was associated with processing the embedded words in the VWFA and other non-visual language-related brain areas.

### ROI analyses

**Fig 3:**
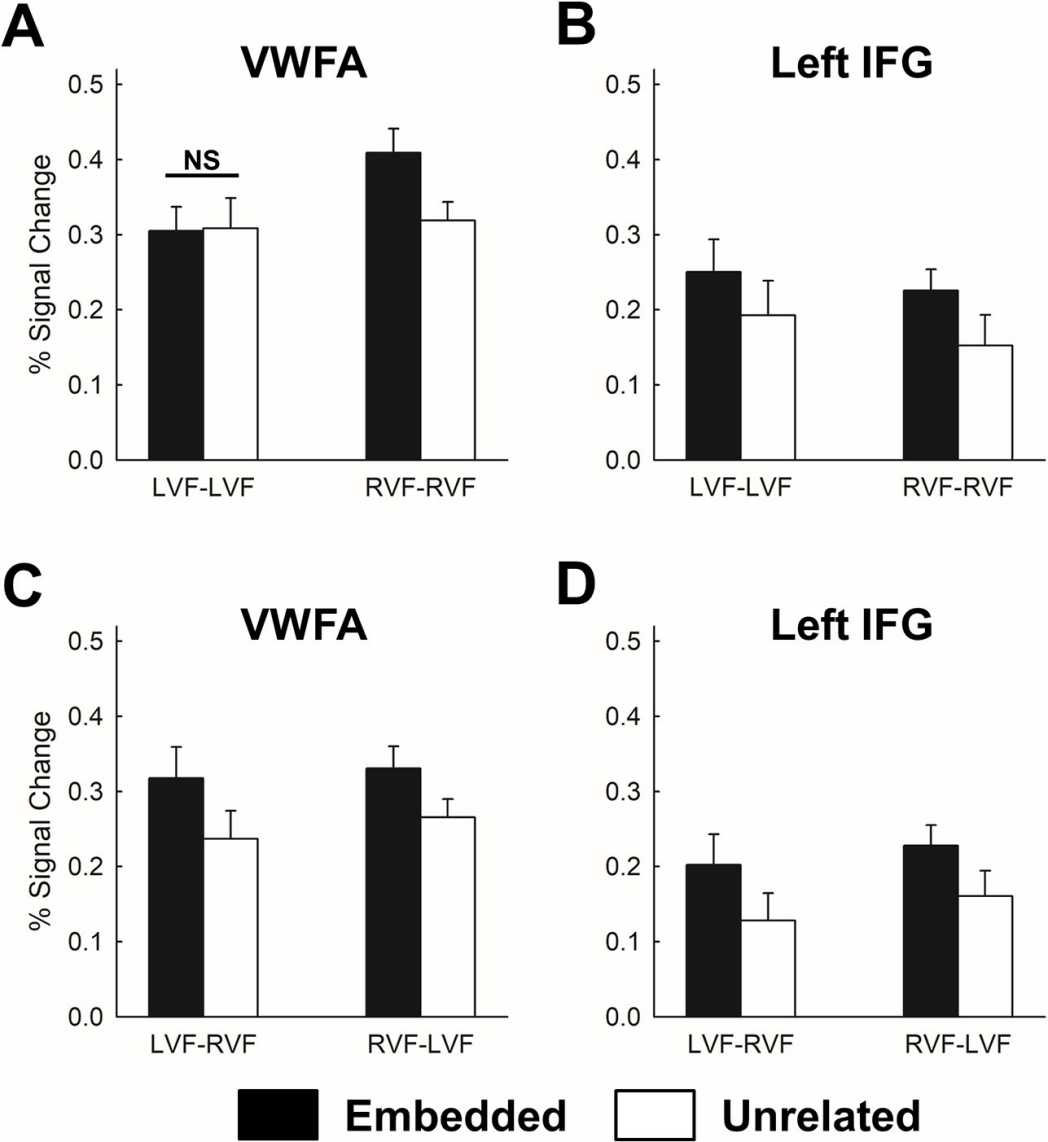
Results of ROI analyses from Experiment 1 and 2. (A) In Experiment 1, the VWFA shows fMRI repetition enhancement for *embedded* words as compared to the *unrelated* words, in Experiment 1. Importantly, the VWFA shows an interaction between the embeddedness and prime-target hemifield, and an absence of fMRI repetition enhancement is observed for the *LVF-LVF* condition (marked by “NS”, non-significant). (B) The fMRI repetition enhancement is observed in the left IFG in Experiment 1. In Experiment 2, the fMRI repetition enhancement is observed in the VWFA (C) and the left IFG (D).

In each observer, we then identified the VWFA defined as Word > Scrambled image contrast. We successfully identified the VWFA for all observers located in anatomical regions constrained to the FG and inferior occipitotemporal sulcus (Table 2). The average Talairach coordinates of the VWFA was located at x = -41.8 ± 2.7, y = -54.9 ± 8.0, z = -12.3 ± 3.3, which was close to the left FG in the whole-brain analyses and also in the vicinity of previously reported VWFA location [2, 10, 53]. Fig 3A shows the BOLD percent signal change in the VWFA from Experiment 1. We performed a two-way repeated measures ANOVA on the BOLD responses for embeddedness (*Embedded* and *Unrelated*) and prime-target hemifield (*LVF-LVF* and *RVF-RVF*). The result suggested a main effect of embeddedness, *F*(1, 10) = 7.29, *p* = 0.022, and an interaction between these two factors, *F*(1, 10) = 10.21, *p* = 0.0096. The main effect of the prime-target hemifield was marginally significant, *F*(1, 10) = 4.85, *p* = 0.052. Post-hoc paired t-test suggested that the fMRI responses for *Embedded* was larger than these for *Unrelated* in the *RVF-RVF* condition, *t*(10) = 4.46, *p* = 0.0012; however, this was not the case in the *LVF-LVF* condition, *t*(10) = -0.12, *p* = 0.91. In short, the VWFA ROI results were consistent with the whole-brain results which suggested that fMRI repetition enhancement for the embedded words, and additionally, such enhancement was observed only for the *RVF-RVF* condition in Experiment 1.

**Table 2:** Center Talairach coordinates and cluster sizes of individual ROIs for all participants.

Participant	Threshold (uncorrected)	VWFA (left)			Number of Voxels	left IFG			Number of Voxels
		x	y	z		x	y	z	
**P1**	**p < 0.01**	**-47**	**-65**	**-13**	**106**	**-44**	**-5**	**36**	**134**
**P2**	**p < 0.05**	**-46**	**-45**	**-15**	**90**	**-39**	**7**	**23**	**36**
**P3**	**p < 0.01**	**-43**	**-52**	**-14**	**79**	**-43**	**10**	**30**	**92**
**P4**	**p < 0.01**	**-39**	**-47**	**-17**	**85**	**-52**	**-2**	**27**	**105**
**P5**	**p < 0.01**	**-38**	**-51**	**-11**	**59**	**-40**	**7**	**25**	**49**
**P6**	**p < 0.05**	**-42**	**-55**	**-6**	**30**	**-41**	**7**	**29**	**107**
**P7**	**p < 0.05**	**-41**	**-56**	**-9**	**54**	**-37**	**2**	**29**	**58**
**P8**	**p < 0.05**	**-42**	**-44**	**-16**	**84**	**-39**	**1**	**27**	**57**
**P9**	**p < 0.05**	**-41**	**-64**	**-10**	**52**	**-42**	**-3**	**29**	**49**
**P10**	**p < 0.05**	**-40**	**-58**	**-14**	**47**	**-31**	**6**	**30**	**20**
**P11**	**p < 0.05**	**-41**	**-67**	**-10**	**30**	**-35**	**13**	**28**	**46**
**Mean**	** **	**-41.8**	**-54.9**	**-12.3**	**65.1**	**-40.3**	**3.9**	**28.5**	**68.5**
**SD**	** **	**2.7**	**8.0**	**3.3**	**25.2**	**5.4**	**5.7**	**3.3**	**35.5**

The left IFG was identified using the same contrast which yielded an average Talairach coordinates located at x = -40.3 ± 5.4, y = 3.9 ± 5.7, z = 28.5 ± 3.3 (Table 2). This result was consistent with the location of the left IFG from the whole-brain analyses (Table 1) and also close to the location reported in previous studies [36, 37]. Fig 3B shows the BOLD percent signal change in the left IFG from Experiment 1. Similar to the VWFA, a two-way repeated measures ANVOA was performed, and the results revealed a main effect of embeddedness, *F*(1, 10) = 6.11, *p* = 0.033. However, there was no main effect of prime-target hemifield, *F*(1, 10) = 2.59, *p* = 0.14, nor interaction between these two, *F*(1, 10) = 0.13, *p* = 0.72. This means that unlike the VWFA, the repetition enhancement effect in the left IFG was not affected by the prime-target hemifield location(s).

Fig 3C shows the BOLD percent signal change for the VWFA from Experiment 2. As before, a two-way repeated measures ANOVA on BOLD percent signal change in the VWFA was conducted. The result showed a main effect of embeddedness, *F*(1, 10) = 9.55, *p* = 0.011. We did not find any main effect of prime-target hemifield (*F*(1, 10) = 0.43, *p* = 0.53) or interaction between these two (*F*(1, 10) = 0.68, *p* = 0.43). This result means that the fMRI repetition enhancement in the VWFA for the embedded words was not affected by primes and targets being viewed sequentially in opposite hemifields. Fig 3D shows the BOLD percent signal change in the left IFG from Experiment 2. We observed similar fMRI responses in the left IFG as shown in Experiment 1. A two-way repeated measures ANVOA revealed a main effect of embeddedness, *F*(1, 10) = 11.86, *p* = 0.006, but neither the main effect of prime-target hemifield (*F*(1, 10) = 2.72, *p* = 0.13) nor the interaction between these two (*F*(1, 10) = 0.067, *p* = 0.80) was statistically significant. In short, both the VWFA and left IFG showed consistent fMRI repetition enhancement in both experiments, but unlike left IFG, the VWFA did not show repetition enhancement for prime-target word pairs presented in the *LVF-LVF* condition in Experiment 1.

## Discussion

The current study used a novel fMRI embedded word priming paradigm. Our main finding was a repetition enhancement effect, which occurred for prime-target word pairs comprised of embedded words followed by containing words. The enhancement effect was not widespread—it was mostly limited to the VWFA in left vOT and other non-visual language-relate brain areas in the left hemisphere (IFG, MFG and PCun). The effect was consistently strong in the VWFA except for a single prime-target hemifield condition (*LVF-LVF*), in which the VWFA failed to show enhancement. We interpret our findings with respect to behavioral studies of embedded word recognition and functional properties of the VWFA reported in other fMRI studies.

### fMRI repetition enhancement and lexical competition

Our finding of fMRI repetition enhancement in the VWFA is the first of its kind. In contrast to fMRI suppression, which is associated with facilitative priming effects [10, 30, 54, 55], fMRI enhancement is associated with inhibitory priming effects [26, 31–33]. Several behavioral studies of word recognition have shown that embedded words are co-activated, automatically and in parallel, with their carrier words [19, 20, 56], which could plausibly result in fMRI enhancement in the context of fMRI priming [26, 27, 57]. Such co-activation is strong enough to connect with meaning and induce semantic interference that arises from sub-word orthographic activation [19]. Consistent with this, interference due to co-activation of embedded words and their carriers is not limited to semantic level competition, and occurs during silent reading during a lexical decision task [24], and other natural reading situations [58]. This is important because although our study employed passive word viewing in conjunction with a fixation task, it is plausible embedded words and their carriers nevertheless co-activated. Consistent with this, models of word recognition posit that representations of a word and its orthographically similar neighbors are co-activated and compete for representation via mutual inhibition during word recognition, a result supported by the results of lexical interference tasks [21].

Additional support for co-activation of embedded words and their carriers in our study relates to our use of the prime-target paradigm. That is, by using embedded words as primes, this may have increased re-activation of the embedded word in the target. Studies of embedded word recognition using a prime-target paradigm similar to that used here have shown orthographic interference in a lexical decision task [22, 23]. Such findings are consistent with the possibility that, in our study, embedded word primes were re-activated when viewing the target carrier word—that is, co-activation of the embedded and carrier word. This co-activation would likely result in orthographic interference arising from inhibitory connections between embedded words and their carriers related to their status as orthographic neighbors [21, 59]. It should also be noted that embedded words interfere with their carriers irrespective of their position within the carrier (i.e., ‘CAR’ → ‘SCAR’ and ‘CAR’ → ‘CART’ exhibits equivalent orthographic interference, [22]), and that lateral inhibition underlying orthographic interference more generally is observed in both masked and non-masked priming paradigms [60, 61]. Taken together, other studies of embedded word viewing have shown that co-activation and interference of embedded words and their carriers occurs in a variety of different contexts.

Consistent with our findings, fMRI enhancement resulting from lexical competition was recently reported in a related study of sub-word orthographic representation. A study by Pas et al. [25] reported an fMRI enhancement effect using a syllabic masked priming paradigm. In their study, repetition enhancement was interpreted to reflect lexical competition between syllabically overlapping words rather than embedded words. The authors concluded that lexical interference resulted from automatic memory retrieval of the prime [26, 33], which caused the interference effect and corresponding fMRI enhancement. Based on their interpretation of fMRI enhancement, the results of behavioral studies of embedded word recognition, and orthographic sensitivity in the VWFA [2, 9–15], we interpret the observed fMRI enhancement as the result of reactivation of primed embedded words, which interfere with their carrier words during viewing of the target.

A possible criticism of our experiment is that the observed enhancement effect was due to our use of a fixation task in conjunction with our conditions of interest. For example, one might argue that attention is drawn to words in the *embedded* condition more than those in the *unrelated* condition because the former involves only very subtle physical (single letter) changes between word pairs, and draws more attention away from the fixation task, resulting in a corresponding increase in fMRI response. Unfortunately, it is not possible to fully rule out the potential contribution of attention-related factors to our results. It should be noted, however, that a previous fMRI repetition suppression study observed equivalent suppression for fixation-based and stimulus-relevant tasks [62]. Additionally, other fMRI studies showed that the degree of repetition suppression corresponds to the magnitude of stimulus change, with small changes producing suppression similar to no change. For example, Fang et al. [63] reported similar degrees of repetition suppression in early visual cortex for both repetition and small stimulus change conditions compared to a large stimulus change condition when participants performed a fixation task. A similar effect has been shown for faces in extrastriate cortex [64, 65]. These findings are difficult to reconcile with an attention-related account of our results in which small stimulus changes lead to repetition enhancement rather than suppression. We therefore conclude that our novel finding of repetition enhancement for embedded words is not necessarily attention-related.

Also, if our enhancement effect was due solely to differences in the degree of attention employed in our different conditions, then we would expect evidence of the effect in both a bilateral ventral visual cortical word recognition circuit [45], in addition to brain regions commonly associated with capture of attention, such as parietal cortex and the temporal parietal junction [66, 67]. However, our results showed no enhancement in these attention related regions, nor in right vOT. Instead, our results indicate that enhancement in word-selective left hemisphere brain regions only, and an interaction of hemifield and condition, which further complicates an attention-based account of our results. We nevertheless concede that attention could play a role in our results, as in other studies of fMRI repetition enhancement [32, 68].

Lastly, even though we predicted repetition suppression could occur, possibly in regions associated with visual processing, no evidence of repetition suppression was found in whole-brain group and additional ROI analyses of the early visual cortex (S1 Fig). Early visual cortex showed brain activation to words presented in the contralateral visual hemifield, but showed no effect of enhancement or suppression. The study by Pas et al. [25], mentioned earlier, also failed to find repetition suppression accompanying enhancement for syllabic repetition. In their study, they observed repetition suppression only for exact stimulus repetitions, as in Glezer et al. [10], also discussed earlier.

### Hemifield-dependent enhancement in the VWFA

A noteworthy exception to fMRI enhancement in the VWFA in our study was the absence of the effect in the *LVF-LVF* condition (Experiment 1), which did not occur in other brain regions that showed fMRI enhancement. This lack of fMRI enhancement was not due to overall decreased fMRI responses in the *LVF-LVF* condition. Our ROI analyses showed that fMRI responses in the *unrelated* prime-target condition did not differ from those in the *RVF-RVF* condition in Experiment 1, and no significant prime-target cross hemifields effect (*LVF-RVF and RVF-LVF*) in Experiment 2, consistent with hemifield-invariant fMRI responses in this condition. This also means that the lack of fMRI enhancement reflects a lack of increased fMRI response in the *embedded* prime-target condition rather than an increased fMRI response in the *unrelated* prime-target condition. In short, fMRI responses to *unrelated* prime-target pairs showed no effect of hemifield, but the re-activation underlying fMRI enhancement did, but only for one particular condition (*LVF-LVF*).

The absence of fMRI enhancement in the *LVF-LVF* hemifield condition was unexpected, and it is difficult to explain. One plausible interpretation is that it reflects a lack of re-activation of the prime, and a consequent absence of competition between the embedded word and its carrier when viewing the target word. The lack of fMRI enhancement in the *LVF-LVF* condition means that, for orthographic interference to occur, an embedded word needs to appear in the RVF, either as a prime, target or both. This could be due to greater sensitivity to orthographic information in the left hemisphere than in the right, and its relation to the location of a word in the visual field [69–71], possibly in conjunction with differences between the VWFA and other non-visual language centers in the left hemisphere (which we discuss in the next section). Alternatively, it is reasonable to hypothesize that the enhancement observed in the VWFA, and its interaction with word location (RVF/LVF) reflects the distinct underlying mechanisms for processing words in the RVF as compared to the LVF. This possibility is consistent with findings of RVF superiority for holistic word processing and feature-based processing of LVF words [72]. According to this view, the highly similar words are more discriminable in the RVF than the LVF, which is associated with stronger representations for these highly similar words in the RVF. Again, this consistent with our interpretation of our results as indicative of unique word-selective processing for words viewed in the RVF, in contrast to other findings of RVF-LVF invariance in the VWFA [2].

Finally, in the negative syllabic priming fMRI study by Pas et al. [25], the authors reasoned that negative priming would be stronger for RVF stimuli as compared to LVF stimuli given stronger lateral inhibition of lexical competitors in the left hemisphere than in the right. Thus, with respect to embedded words, LVF prime-target viewing could result in decreased lateral inhibition between embedded words and their carriers in addition to either weaker activation of the embedded word prime, its re-activation in the target, or both. Additionally, it is possible that because only the left hemisphere VWFA represents whole words [10], then the right hemisphere fails to activate embedded target words; an analogous argument has been proposed for lack of fMRI repetition effect for LVF viewed face stimuli [73]. The failure of embedded word re-activation could also be due to callosal transfer of LVF words for left hemisphere processing, which results in a time delay and reduction of quality of stimulus representation [74]. Alternatively, it is also possible that the repetition enhancement may be offset by repetition suppression in *LVF-LVF* condition. For instance, it has been shown that orthographic processing was associated with facilitation for words presented in the LVF, at the sublexical level of orthographic coding [75], but inhibition for words presented in the RVF [76]. Thus, presenting the embedded words twice in the LVF may lead to a combined facilitative effect of sublexical repetition between primes and targets, and lexical competition between whole-word representations of the primes and the targets.

### Beyond the VWFA

Although our primary brain region of interest was the VWFA, our results revealed a dissociation between the VWFA and other non-visual brain areas implicated elsewhere in orthographic processing, including left IFG. Early studies have shown the involvement of the left IFG in sematic and phonological processing (for a review see [77]), but recent studies have shifted attention to the role of the left IFG in orthographic processing [36, 37]. Our results thus offer further evidence of orthographic representation in left IFG. Consistent with this, left IFG has been shown involving higher-level abstract orthographic processing [78] and orthographic long-term memory [79]. In the fMRI study by Pas et al. [25], discussed earlier, the authors argued that the observed repetition enhancement in the left vOT (likely the VWFA) was driven by the feedback responses from the left IFG, which acts as a fast visual word processing system [80, 81], faster than and distinct from the VWFA. This distinction may explain the lack of hemifield effect on fMRI enhancement observed in the left IFG in our study. It should be noted that the aim of the current study was not to separate effects related to orthographic or semantic levels processed in different brain regions. It is however possible that, unlike the VWFA which only showed orthographic sensitivity [14], the left IFG could be associated with semantic processing, consistent with the view that this region is involved in the neural representation of competing semantic information [82]. This conclusion is tentative because of our fixation task instead of a semantic categorization task, which would allow for a behavioral measure of semantic interference (e.g., [19]).

In addition to left IFG, our whole-brain analyses also show fMRI enhancement in left PCun and left MFG. Unlike left IFG, these areas were not identified in our independent localizer and we were therefore unable to perform ROI analyses as we did for left IFG. Both areas are commonly activated in fMRI studies of word recognition and reading. Left PCun has been implicated in orthographic representation [83] and monitoring the orthographic and phonological consistency related to attention [84], however, it is also has been suggested to associate with semantic representation [85]. A large amount of studies have suggested that left MFG is also associated with semantic representation (for a review see [77]), in addition to allocation spatial attention during word recognition [86], but it is not strongly associated with orthographic representation. It is possible that our finding of repetition enhancement in left PCun and left MFG is related to the co-activation of semantic representations of the embedded words, consistent with the view that sub-word orthographic interference is strong enough to connect with meaning and induce semantic interference [20, 87]. However, this interpretation could not be tested in the current study, because we did not control for the semantic relationship between words in the *embedded* and *unrelated* conditions.

In conclusion, the present study provides the first neural evidence of lexical interference during embedded word viewing. Observation of the fMRI repetition enhancement in the VWFA and its failure under the *LVF-LVF* prime-target hemifield condition revealed a clear dissociation between the VWFA and non-visual language-related areas in the brain. Our findings are consistent with the view that the VWFA underlies lexical-level orthographic representation. Our findings also support the view that the VWFA is distinct from other brain areas involved in orthographic neural representation, and which possibly exert feedback effects on the VWFA.

## Supporting information

S1 Table Word list used in experiment 1 and 2. (DOCX)

S1 Fig BOLD responses in early visual areas for Experiment 1 for 7 (of the 11) subjects. V1 and V2/3 combined were defined using standard retinotopic mapping procedures. Both, in each hemisphere, showed strong contralateral bias to word pairs presented in either the LVF or RVF but no evidence of BOLD differences between the *embedded* and *unrelated* conditions matched by LVF/RVF.
(TIF)
