# Supplementary material for: Embedded word priming elicits enhanced fMRI responses in the visual word form area
Source: PLoS One. 2019 Jan 10;14(1):e0208318. doi: 10.1371/journal.pone.0208318 (PMC6328158; doi:10.1371/journal.pone.0208318)
Supplement: S1 Table — (DOCX) [file pone.0208318.s001.docx]

**S1 Table. Word list used in experiment 1 and 2.**

| First letter addition | | | Last letter addition | | |
| --- | --- | --- | --- | --- | --- |
| Embedded | Carrier | Unrelated | Embedded | Carrier | Unrelated |
| ACE | FACE | FONT | ARC | ARCH | GOSH |
| ACT | PACT | PING | BAD | BADE | WILE |
| AFT | RAFT | RICH | BAR | BARK | SOCK |
| AGE | WAGE | WOMB | BAT | BATH | MESH |
| AID | RAID | ROVE | BID | BIDE | ALOE |
| AIL | TAIL | TECH | BIN | BING | FROG |
| AIR | HAIR | HULK | BIT | BITE | VASE |
| AMP | RAMP | RENT | BOA | BOAR | EMIR |
| AND | WAND | WIRE | BOD | BODE | TYPE |
| ANT | RANT | RILE | BOW | BOWL | REAL |
| ARE | CARE | COUP | BRA | BRAY | PITY |
| ARK | PARK | PUNT | BUM | BUMP | SWAP |
| ARM | WARM | WISP | BUN | BUNK | HECK |
| ART | PART | PINK | BUS | BUSY | ONLY |
| ASH | CASH | CURL | CAM | CAMP | BLIP |
| ASK | TASK | THIN | CAP | CAPE | GIVE |
| ATE | RATE | ROMP | CHI | CHIN | FAWN |
| CAB | SCAB | SMUT | COD | CODE | SAME |
| CAN | SCAN | SLOT | CON | CONE | FAME |
| CAR | SCAR | STEM | COP | COPY | TIDY |
| CAT | SCAT | SNUG | CUB | CUBE | LIFE |
| CUD | SCUD | STEW | CUT | CUTE | NAME |
| EAT | MEAT | MORN | DAM | DAME | LUTE |
| ELF | SELF | SNOB | DEN | DENT | GAIT |
| ELM | HELM | HUNG | DIE | DIET | PAST |
| END | REND | RUSK | DIM | DIME | ABLE |
| ETA | BETA | BIND | DIN | DINE | ACRE |
| HAG | SHAG | SLOP | DUE | DUEL | OPAL |
| HAM | SHAM | SNOW | DUN | DUNK | HICK |
| HEN | WHEN | WORK | EAR | EARN | SIGN |
| HEW | PHEW | PAIL | FAD | FADE | SIZE |
| HIM | WHIM | WAKE | FAN | FANG | CHUG |
| HIP | SHIP | SLOW | FAR | FARM | PLUM |
| HIS | THIS | TEMP | FAT | FATE | WIDE |
| HOE | SHOE | SULK | FIN | FINE | LODE |
| HOT | SHOT | SANE | FIR | FIRM | TEAM |
| HOW | CHOW | CARD | FLU | FLUX | COAX |
| HUT | SHUT | SOFA | FOR | FORE | WAVE |
| ICE | LICE | LAST | FUN | FUND | VOID |
| ILK | SILK | SOME | FUR | FURY | PLAY |
| IMP | LIMP | LOUD | GAL | GALE | CITE |
| INK | RINK | RUST | GAS | GASH | OUCH |
| ION | LION | LACK | GUN | GUNK | BACK |
| IRE | TIRE | THUD | HAS | HAST | DOLT |
| KID | SKID | SALT | HAT | HATE | BORE |
| LAB | SLAB | SPUR | HAW | HAWK | DESK |
| LAG | SLAG | SORT | HEM | HEMP | FLIP |
| LAP | SLAP | SUIT | HER | HERD | WILD |
| LAX | FLAX | FEND | HID | HIDE | VOTE |
| LAY | CLAY | COST | HOP | HOPE | LACE |
| LED | BLED | BUOY | HUG | HUGE | MOVE |
| LID | SLID | SPAM | HUM | HUMP | STEP |
| LIP | SLIP | SAFE | KIN | KIND | CORD |
| LIT | SLIT | SUNK | KIT | KITE | MORE |
| LOG | FLOG | FEAR | LAD | LADY | RUBY |
| LOP | FLOP | FEUD | LAW | LAWN | GRIN |
| LOT | PLOT | PAIR | LIE | LIEN | YAWN |
| LOW | PLOW | PATH | LOB | LOBE | MAZE |
| LUG | PLUG | PONY | MAD | MADE | LORE |
| MUG | SMUG | SOAP | MAN | MANY | CITY |
| NAG | SNAG | SOUP | MAR | MARE | YOKE |
| NAP | SNAP | STIR | MAT | MATH | JOSH |
| NIP | SNIP | SLED | MAY | MAYO | HERO |
| NOW | KNOW | KEPT | MEN | MEND | WARD |
| NUB | SNUB | SAIL | MOP | MOPE | HIVE |
| OAF | LOAF | LEWD | NEW | NEWT | KILT |
| OAK | SOAK | SURE | NOD | NODE | PYRE |
| OAR | SOAR | SPUD | NOR | NORM | EXAM |
| ODE | MODE | MAIL | NOT | NOTE | SAVE |
| OIL | COIL | CZAR | OAT | OATH | INCH |
| OLD | BOLD | BANK | PAL | PALM | GRIM |
| ONE | BONE | BALM | PAN | PANT | WRIT |
| ORE | WORE | WASH | PAW | PAWN | JOIN |
| OUR | FOUR | FLEX | PEA | PEAK | TUCK |
| OUT | GOUT | GIRL | PER | PERM | SWIM |
| OWL | HOWL | HINT | PIE | PIED | FOND |
| OWN | GOWN | GLIB | PRO | PROP | WASP |
| PAR | SPAR | SHIN | ROB | ROBE | PAVE |
| PAT | SPAT | SKIN | RUN | RUNE | TIME |
| PEW | SPEW | STUN | SAC | SACK | PORK |
| PIT | SPIT | SAND | SAG | SAGE | TORE |
| POT | SPOT | SWAM | SEA | SEAM | GLUM |
| PRY | SPRY | SNOT | SEC | SECT | FLAT |
| PUN | SPUN | SILT | SHE | SHED | BALD |
| PUS | OPUS | ORAL | SIC | SICK | TREK |
| RAG | DRAG | DUMB | SIN | SINK | MACK |
| RAM | TRAM | TOIL | SIR | SIRE | GAZE |
| RAN | BRAN | BOCK | SIT | SITE | RACE |
| RAT | DRAT | DUNG | SKI | SKIP | BURP |
| RAW | DRAW | DOPE | SOD | SODA | HULA |
| RAY | PRAY | PONG | SON | SONG | THUG |
| RED | BRED | BOUT | SPA | SPAN | HYMN |
| RIB | CRIB | COLA | SUN | SUNG | CRAG |
| RID | ARID | ANEW | TAN | TANK | HOCK |
| RIG | BRIG | BEAU | TAP | TAPE | LINE |
| RIM | PRIM | PACE | TAX | TAXI | SEMI |
| RIP | GRIP | GULF | TEA | TEAL | SOIL |
| ROD | PROD | PANE | TEN | TEND | LOAD |
| ROW | CROW | CLAM | TIC | TICK | SANK |
| RUB | GRUB | GLAD | TIE | TIER | BLUR |
| RUG | DRUG | DEAL | TIN | TINY | GRAY |
| TAB | STAB | SORE | TON | TONG | BRAG |
| TAG | STAG | SPED | VAN | VANE | CURE |
| TAR | STAR | SKIM | VET | VETO | KILO |
| TOP | STOP | SWAB | VIA | VIAL | FOUL |
| TOW | STOW | SALE | VIE | VIEW | BROW |
| TUB | STUB | SLEW | WAD | WADE | ROSE |
| URN | TURN | TOMB | WAR | WARE | POLE |
| USE | MUSE | MINT | WAS | WASP | KELP |
| WAG | SWAG | SILO | WIN | WING | SMOG |
| WAY | SWAY | SING | WIT | WITH | BASH |
| WIG | SWIG | SEND | WON | WONT | FELT |
