# Supplementary figures and images for: Embedded word priming elicits enhanced fMRI responses in the visual word form area
Source: PLoS One. 2019 Jan 10;14(1):e0208318. doi: 10.1371/journal.pone.0208318 (PMC6328158; doi:10.1371/journal.pone.0208318)

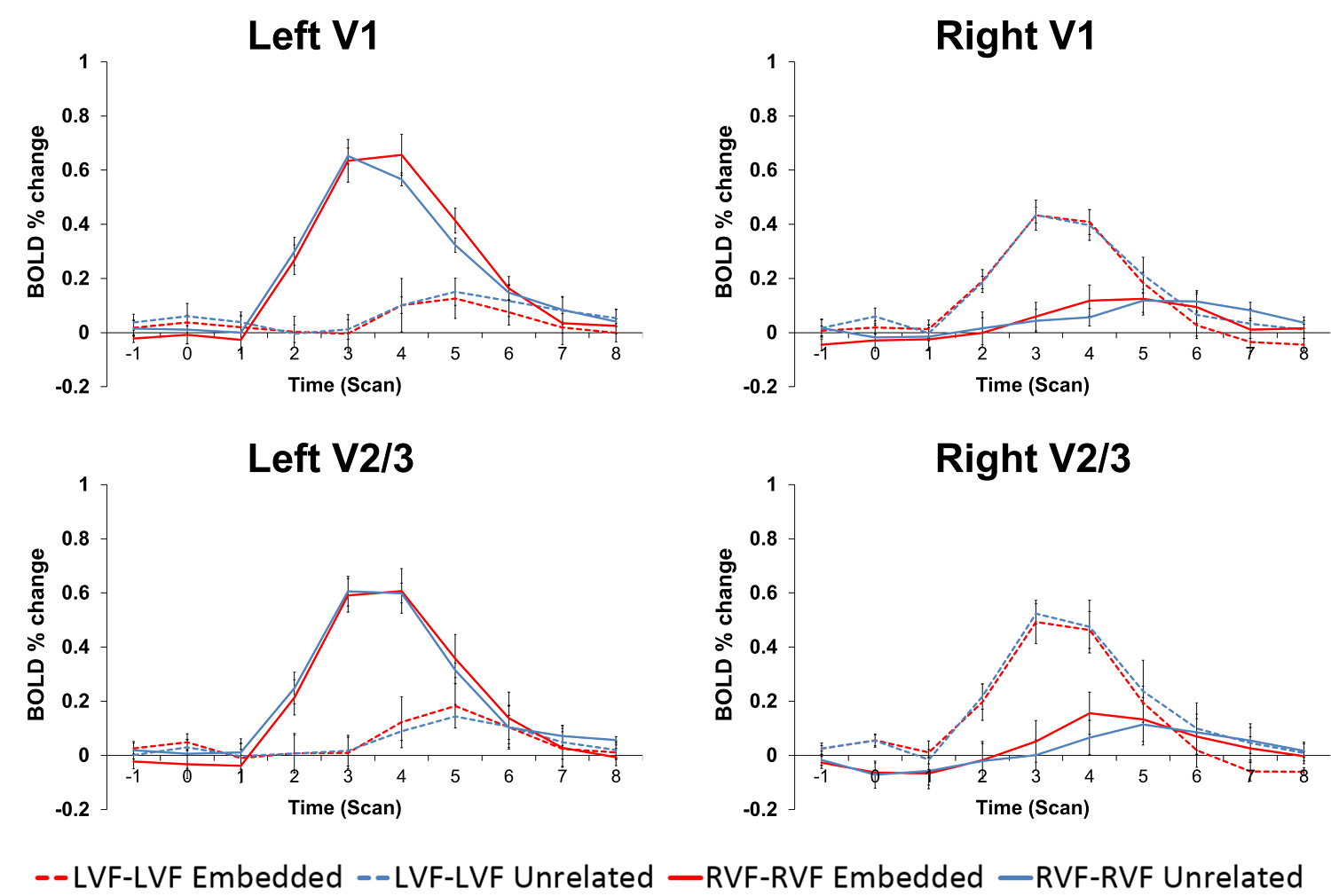

Supplement: S1 Fig — V1 and V2/3 combined were defined using standard retinotopic mapping procedures. Both, in each hemisphere, showed strong contralateral bias to word pairs presented in either the LVF or RVF but no evidence of BOLD differences between the embedded and unrelated conditions matched by LVF/RVF. (TIF) [file pone.0208318.s002.tif]
